# Supplementary material for: Biological Hazards and Indicators Found in Products of Animal Origin in Cambodia from 2000 to 2022: A Systematic Review
Source: Int J Environ Res Public Health. 2024 Dec 3;21(12):1621. doi: 10.3390/ijerph21121621 (PMC11675544; doi:10.3390/ijerph21121621)
Supplement: Supplementary file 1 [file ijerph-21-01621-s001.zip › ijerph-3242238-supplementary.pdf]

Supplementary material S1

Definitions of the key terms used in the review

Hazard- A biological, chemical, or physical agent in food with the potential to cause an adverse health effect. (Codex Alimentarius Commission of FAO and WHO, 2020)

Outcome- An event or measurement collected for participants in a study such as quality of life, mortality (Page et.al., 2021).

Protocol- In the context of systematic reviews and meta-analyses, a protocol is a document that presents an explicit plan for a systematic review. The protocol details the rationale and a priori methodological and analytical approach of the review (Page et.al., 2021).

Result- The combination of a point estimate (such as a mean difference, risk ratio, or proportion) and a measure of its precision (such as a confidence/credible interval) for a particular outcome (Page et.al., 2021).

Report- A document (paper or electronic) supplying information about a particular study. It could be a journal article, preprint, conference abstract, study register entry, clinical study report, dissertation, unpublished manuscript, government report, or any other document providing relevant information (Page et.al., 2021).

Record -The title or abstract (or both) of a report indexed in a database or website such as a title or abstract for an article indexed in Medline (Page et.al., 2021).

Records that refer to the same report (such as the same journal article) are “duplicates”; however, records that refer to reports that are merely similar (such as a similar abstract submitted to two different conferences) should be considered unique (Page et.al., 2021).

Study- An investigation, such as a clinical trial, that includes a defined group of participants and one or more interventions and outcomes. A “study” might have multiple reports (Page et.al., 2021).

Systematic review- A review that uses explicit, systematic methods to collate and synthesise findings of studies that address a clearly formulated question (Page et.al., 2021).

PROSPERO- International Prospective Register of Systematic Reviews

PROSPERO is an internet platform where researchers can register their plans to conduct a systematic review with health-related outcomes before commencing the study. The key objective of PROSPERO is to ensure that the intention to conduct systematic reviews is made known in advance, thereby minimizing the occurrence of unintentional duplications. Furthermore, the register promotes greater transparency in the review process by mandating the documentation of pre-planned methods. This enables readers of systematic reviews to compare the methods, outcomes, and analyses conducted with those originally intended, and assess the potential impact of any modifications on the review's findings (Moher et al., 2015).

PRISMA-P- Preferred Reporting Items for Systematic Review and Meta-Analysis Protocols

PRISMA-P is a guide to assist authors in preparing protocols for planned systematic reviews and meta-analyses. The guide includes a minimum set of items that should be included in the protocol. The purpose of the protocol is to provide the rationale for the review and outline the planned methodology and analysis. Authors are advised to prepare the protocol before registering it in PROSPERO to avoid the need for multiple amendments. The PRISMA-P items are based on the PRISMA checklist and PROSPERO register to ensure the smooth and straightforward registration (Moher et al., 2015).

Supplementary Table S2

Studies included in the review

Paper No.	Reference
**1**	Tum, S.; Puotinen, M. L.; Copeman, D. B. A Geographic Information Systems Model for Mapping Risk of Fasciolosis in Cattle and Buffaloes in Cambodia. Vet Parasitol 2004, 122 (2), 141–149. https://doi.org/10.1016/j.vetpar.2004.03.016.
**2**	Than Sovyra. Prevalence of Porcine Cysticercosis and Trichinellosis in Slaughter Pigs of Cambodia. , Thailand: Chiang Mai University., 2005. [online] repository.cmu.ac.th. Available at: http://repository.cmu.ac.th/handle/6653943832/35803 [Accessed 20 Mar. 2023].
**3**	Siengsanan-Lamont, J.; Tum, S.; Kong, L.; Selleck, P. W.; Gleeson, L. J.; Blacksell, S. D. Abattoir-Based Serological Surveillance for Transboundary and Zoonotic Diseases in Cattle and Swine in Cambodia: A Pilot Study in Phnom Penh Province during 2019 and 2020. Trop Anim Health Prod 2022, 54 (5), 316. https://doi.org/10.1007/s11250-022-03309-1.
**4**	Vuthy, Y.; Lay, K. S.; Seiha, H.; Kerleguer, A.; Aidara-Kane, A. Antibiotic Susceptibility and Molecular Characterization of Resistance Genes among Escherichia Coli and among Salmonella Subsp. in Chicken Food Chains. Asian Pac J Trop Biomed 2017, 7 (7), 670–674. https://doi.org/10.1016/j.apjtb.2017.07.002.
**5**	Loeurng, V.; Chea, B.; Tum, S.; Seng, M. Challenge and Prevalence of Fasciolosis in Cattle in Pursat Province, Cambodia. IJERD – International Journal of Environmental and Rural Development 2016, 7 (1). [online] *IJERD -International Journal of Environmental and Rural Development*, pp.7–8. Available at: https://iserd.net/ijerd71/IJERD%207-1-11.pdf.
**6**	Nadimpalli, M.; Fabre, L.; Yith, V.; Sem, N.; Gouali, M.; Delarocque-Astagneau, E.; Sreng, N.; Le Hello, S.; Raheliarivao, B. T.; Randrianirina, F.; Herindrainy, P.; Andrianirina, Z. Z.; Rakotoarimanana, F. M. J.; Garin, B.; Collard, J.-M.; de Lauzanne, A.; Borand, L.; Piola, P.; Kerléguer, A.; Chon, T.; Touch, S.; Tarantola, A.; Goyet, S.; Lach, S.; Ngo, V.; Vray, M.; Diatta, M.; Faye, J.; Ndiaye, A.; Richard, V.; Seck, A.; Bercion, R.; Sow, A. G.; Diouf, J. B.; Dieye, P. S.; Sy, B.; Ndao, B.; Guillemot, D.; Huynh, B.; Seguy, M.; Watier, L.; Youssouf, A. A.; Padget, M. CTX-M-55-Type ESBL-Producing *Salmonella Enterica* Are Emerging among Retail Meats in Phnom Penh, Cambodia. Journal of Antimicrobial Chemotherapy 2019, 74 (2), 342–348. https://doi.org/10.1093/jac/dky451.
**7**	Wang, W.; Owen, H.; Traub, R. J.; Cuttell, L.; Inpankaew, T.; Bielefeldt-Ohmann, H. Molecular Epidemiology of Blastocystis in Pigs and Their In-Contact Humans in Southeast Queensland, Australia, and Cambodia. Vet Parasitol 2014, 203 (3–4), 264–269. https://doi.org/10.1016/j.vetpar.2014.04.006.

**8**	Osbjer, K.; Tano, E.; Chhayheng, L.; Mac‐Kwashie, A. O.; Fernström, L.; Ellström, P.; Sokerya, S.; Sokheng, C.; Mom, V.; Chheng, K.; San, S.; Davun, H.; Boqvist, S.; Rautelin, H.; Magnusson, U. Detection of Campylobacter in Human and Animal Field Samples in Cambodia. APMIS 2016, 124 (6), 508–515. https://doi.org/10.1111/apm.12531.
**9**	Sohn, W.-M.; Jung, B.-K.; Hong, S.; Ryoo, S.; Lee, K. H.; Khieu, V.; Chai, J.-Y. Detection of Gnathostoma Spinigerum Advanced 3rd-Stage Larvae in the Chinese Edible Frog, Hoplobatrachus Rugulosus, from Local Markets in Phnom Penh, Cambodia. Korean J Parasitol 2021, 59 (5), 519–522. https://doi.org/10.3347/kjp.2021.59.5.519.
**10**	Touch, S.; Komalamisra, C.; Radomyos, P.; Waikagul, J. Discovery of Opisthorchis Viverrini Metacercariae in Freshwater Fish in Southern Cambodia. Acta Trop 2009, 111 (2), 108–113. https://doi.org/10.1016/j.actatropica.2009.03.002.
**11**	Lacroix, A.; Duong, V.; Hul, V.; San, S.; Davun, H.; Omaliss, K.; Chea, S.; Hassanin, A.; Theppangna, W.; Silithammavong, S.; Khammavong, K.; Singhalath, S.; Afelt, A.; Greatorex, Z.; Fine, A. E.; Goldstein, T.; Olson, S.; Joly, D. O.; Keatts, L.; Dussart, P.; Frutos, R.; Buchy, P. Diversity of Bat Astroviruses in Lao PDR and Cambodia. Infection, Genetics and Evolution 2017, 47, 41–50. https://doi.org/10.1016/j.meegid.2016.11.013.
**12**	Chai, J.-Y.; Sohn, W.-M.; Cho, J.; Jung, B.-K.; Chang, T.; Lee, K. H.; Khieu, V.; Huy, R. Echinostoma Mekongi: Discovery of Its Metacercarial Stage in Snails, Filopaludina Martensi Cambodjensis, in Pursat Province, Cambodia. Korean J Parasitol 2021, 59 (1), 47–53. https://doi.org/10.3347/kjp.2021.59.1.47.
**13**	Miyamoto, K.; Kirinoki, M.; Matsuda, H.; Hayashi, N.; Chigusa, Y.; Sinuon, M.; Chuor, C. M.; Kitikoon, V. Field Survey Focused on Opisthorchis Viverrini Infection in Five Provinces of Cambodia. Parasitol Int 2014, 63 (2), 366–373. https://doi.org/10.1016/j.parint.2013.12.003.
**14**	Rodriguez, C.; Mith, H.; Taminiau, B.; Bouchafa, L.; Van Broeck, J.; Soumillion, K.; Ngyuvula, E.; García-Fuentes, E.; Korsak, N.; Delmée, M.; Daube, G. First Isolation of Clostridioides Difficile from Smoked and Dried Freshwater Fish in Cambodia. Food Control 2021, 124, 107895. https://doi.org/10.1016/j.foodcont.2021.107895.
**15**	Touch, S. ,; Tippayarat Yoonuan; Supaporn Nuamtanong; Nirandorn Homsuwan; Orawan Phuphisut; Thaenkham, U. ,; Waikagul, J. Seasonal Variation of Opisthorchis Viverrini Metacercarial Infection in Cyprinid Fish from Southern Cambodia. The Journal of Tropical Medicine and Parasitology 2013, 36 (1), 1–7.
**16**	Dorny, P.; Stoliaroff, V.; Charlier, J.; Meas, S.; Sorn, S.; Chea, B.; Holl, D.; Van Aken, D.; Vercruysse, J. Infections with Gastrointestinal Nematodes, Fasciola and Paramphistomum in Cattle in Cambodia and Their Association with Morbidity Parameters. Vet Parasitol 2011, 175 (3–4), 293–299. https://doi.org/10.1016/j.vetpar.2010.10.023.

**17**	Lv, S.; Guo, Y.-H.; Nguyen, H. M.; Sinuon, M.; Sayasone, S.; Lo, N. C.; Zhou, X.-N.; Andrews, J. R. Invasive Pomacea Snails as Important Intermediate Hosts of Angiostrongylus Cantonensis in Laos, Cambodia and Vietnam: Implications for Outbreaks of Eosinophilic Meningitis. Acta Trop 2018, 183, 32–35. https://doi.org/10.1016/j.actatropica.2018.03.021.
**18**	Cappelle, J.; Hoem, T.; Hul, V.; Furey, N.; Nguon, K.; Prigent, S.; Dupon, L.; Ken, S.; Neung, C.; Hok, V.; Pring, L.; Lim, T.; Bumrungsri, S.; Duboz, R.; Buchy, P.; Ly, S.; Duong, V.; Tarantola, A.; Binot, A.; Dussart, P. Nipah Virus Circulation at Human–Bat Interfaces, Cambodia. Bull World Health Organ 2020, 98 (8), 539–547. https://doi.org/10.2471/BLT.20.254227.
**19**	Reynes, J.-M.; Counor, D.; Ong, S.; Faure, C.; Seng, V.; Molia, S.; Walston, J.; Georges-Courbot, M. C.; Deubel, V.; Sarthou, J.-L. Nipah Virus in Lyle's Flying Foxes, Cambodia. Emerg Infect Dis 2005, 11 (7), 1042–1047. https://doi.org/10.3201/eid1107.041350.
**20**	Trongjit, S.; Angkittitrakul, S.; Chuanchuen, R. Occurrence and Molecular Characteristics of Antimicrobial Resistance of Escherichia Coli from Broilers, Pigs and Meat Products in Thailand and Cambodia Provinces. Microbiol Immunol 2016, 60 (9), 575–585. https://doi.org/10.1111/1348-0421.12407.
**21**	Hongchuta, S.; Intapan, P. M.; Thanchomnang, T.; Sadaow, L.; Rodpai, R.; Laummuanwai, P.; Maleewong, W.; Sanpool, O. Preliminary Findings and Molecular Characterization of Thin-Walled Sarcocystis Species in Hearts of Cattle and Buffaloes in Thailand, Lao PDR, and Cambodia. Parasitol Res 2021, 120 (8), 2819–2825. https://doi.org/10.1007/s00436-021-07241-5.
**22**	Trongjit, S.; Angkititrakul, S.; Tuttle, R. E.; Poungseree, J.; Padungtod, P.; Chuanchuen, R. Prevalence and Antimicrobial Resistance in Salmonella Enterica Isolated from Broiler Chickens, Pigs and Meat Products in Thailand–Cambodia Border Provinces. Microbiol Immunol 2017, 61 (1), 23–33. https://doi.org/10.1111/1348-0421.12462.
**23**	Sohn, W.-M.; Choi, S.-H.; Jung, B.-K.; Hong, S.; Ryoo, S.; Chang, T.; Lee, K. H.; Na, B.-K.; Hong, S.-J.; Khieu, V.; Chai, J.-Y. Prevalence and Intensity of Opisthorchis Viverrini Metacercarial Infection in Fish from Phnom Penh, Takeo, and Kandal Provinces, Cambodia. Korean J Parasitol 2021, 59 (5), 531–536. https://doi.org/10.3347/kjp.2021.59.5.531.
**24**	Sohn, W.-M.; Yong, T.-S.; Eom, K. S.; Pyo, K.-H.; Lee, M. Y.; Lim, H.; Choe, S.; Jeong, H.-G.; Sinuon, M.; Socheat, D.; Chai, J.-Y. Prevalence of Opisthorchis Viverrini Infection in Humans and Fish in Kratie Province, Cambodia. Acta Trop 2012, 124 (3), 215–220. https://doi.org/10.1016/j.actatropica.2012.08.011.
**25**	Rortana, C.; Nguyen-Viet, H.; Tum, S.; Unger, F.; Boqvist, S.; Dang-Xuan, S.; Koam, S.; Grace, D.; Osbjer, K.; Heng, T.; Sarim, S.; Phirum, O.; Sophia, R.; Lindahl, J. F. Prevalence of

	Salmonella Spp. and Staphylococcus Aureus in Chicken Meat and Pork from Cambodian Markets. Pathogens 2021, 10 (5), 556. https://doi.org/10.3390/pathogens10050556.
**26**	Lay, K. S.; Vuthy, Y.; Song, P.; Phol, K.; Sarthou, J. L. Prevalence, Numbers and Antimicrobial Susceptibilities of Salmonella Serovars and Campylobacter Spp. in Retail Poultry in Phnom Penh, Cambodia. Journal of Veterinary Medical Science 2011, 73 (3), 325–329. https://doi.org/10.1292/jvms.10-0373
**27**	Sohn, W.-M.; Kim, D.-G.; Jung, B.-K.; Cho, J.; Chai, J.-Y. Pygidiopsis Cambodiensis n. Sp. (Digenea: Heterophyidae) from Experimental Hamsters Infected with Metacercariae in Mullets from Cambodia. Parasitol Res 2016, 115 (1), 123–130. https://doi.org/10.1007/s00436-015-4727-1.
**28**	Adenuga, A.; Mateus, A.; Ty, C.; Borin, K.; Holl, D.; San, S.; Duggan, V.; Clark, M.; Smith, G. J. D.; Coker, R.; Vaughn, A.; Rudge, J. W. Seroprevalence and Awareness of Porcine Cysticercosis across Different Pig Production Systems in South-Central Cambodia. Parasite Epidemiol Control 2018, 3 (1), 1–12. https://doi.org/10.1016/j.parepi.2017.10.003.
**29**	Chai, J.-Y.; Sohn, W.-M.; Na, B.-K.; Jeoung, H.-G.; Sinuon, M.; Socheat, D. Stellantchasmus Falcatus (Digenea: Heterophyidae) in Cambodia: Discovery of Metacercariae in Mullets and Recovery of Adult Flukes in an Experimental Hamster. Korean J Parasitol 2016, 54 (4), 537–541. https://doi.org/10.3347/kjp.2016.54.4.537.
**30**	Söderberg, R.; Lindahl, J. F.; Henriksson, E.; Kroesna, K.; Ly, S.; Sear, B.; Unger, F.; Tum, S.; Nguyen-Viet, H.; Ström Hallenberg, G. Low Prevalence of Cysticercosis and Trichinella Infection in Pigs in Rural Cambodia. Trop Med Infect Dis 2021, 6 (2), 100. https://doi.org/10.3390/tropicalmed6020100.
**31**	Chai, J.-Y.; Jung, B.-K.; Lee, K. H.; Hong, S.-J.; Khieu, V.; Na, B.-K.; Sohn, W.-M. Infection Status of Gnathostoma Spinigerum Larvae in Asian Swamp Eels, Monopterus Albus, Purchased from Local Markets in Cambodia. Korean J Parasitol 2020, 58 (6), 695–699. https://doi.org/10.3347/kjp.2020.58.6.695.
**32**	Nadimpalli, M.; Vuthy, Y.; de Lauzanne, A.; Fabre, L.; Criscuolo, A.; Gouali, M.; Huynh, B.-T.; Naas, T.; Phe, T.; Borand, L.; Jacobs, J.; Kerléguer, A.; Piola, P.; Guillemot, D.; Le Hello, S.; Delarocque-Astagneau, E. Meat and Fish as Sources of Extended-Spectrum β-Lactamase–Producing Escherichia Coli , Cambodia. Emerg Infect Dis 2019, 25 (1). https://doi.org/10.3201/eid2501.180534.
**33**	Sohn, W.-M.; Yong, T.-S.; Eom, K. S.; Sinuon, M.; Jeoung, H.-G.; Chai, J.-Y. Artyfechinostomum Malayanum: Metacercariae Encysted in Pila Sp. Snails Purchased from Phnom Penh, Cambodia. Korean J Parasitol 2017, 55 (3), 341–345. https://doi.org/10.3347/kjp.2017.55.3.341.

**34**	Boonmekam, D. , N. S. , M. H. , K. M. , M. K. , S. M. , & K. D. Morphological and Molecular Identification of the Liver Fluke Opisthorchis Viverrini in the First Intermediate Host Bithynia Snails and Its Prevalence in Kampong Cham Province, Cambodia. . Parasitology International 2017, 66 (3), 319–323. doi:https://doi.org/10.1016/j.parint.2017.01.016.
**35**	Schär, F.; Inpankaew, T.; Traub, R. J.; Khieu, V.; Dalsgaard, A.; Chimnoi, W.; Chhoun, C.; Sok, D.; Marti, H.; Muth, S.; Odermatt, P. The Prevalence and Diversity of Intestinal Parasitic Infections in Humans and Domestic Animals in a Rural Cambodian Village. Parasitol Int 2014, 63 (4), 597–603. https://doi.org/10.1016/j.parint.2014.03.007.
**36**	Chai, J.-Y.; Sohn, W.-M.; Na, B.-K.; Yong, T.-S.; Eom, K. S.; Yoon, C.-H.; Hoang, E.-H.; Jeoung, H.-G.; Socheat, D. Zoonotic Trematode Metacercariae in Fish from Phnom Penh and Pursat, Cambodia. Korean J Parasitol 2014, 52 (1), 35–40. https://doi.org/10.3347/kjp.2014.52.1.35.
**37**	Caron, M.; Enouf, V.; Than, S. C.; Dellamonica, L.; Buisson, Y.; Nicand, E. Identification of Genotype 1 Hepatitis E Virus in Samples from Swine in Cambodia. J Clin Microbiol 2006, 44 (9), 3440–3442. https://doi.org/10.1128/JCM.00939-06.
**38**	Olson, J. G.; Rupprecht, C.; Rollin, P. E.; An, U. S.; Niezgoda, M.; Clemins, T.; Walston, J.; Ksiazek, T. G. Antibodies to Nipah-Like Virus in Bats (Pteropus Lylei) , Cambodia. Emerg Infect Dis 2002, 8 (9), 987–988. https://doi.org/10.3201/eid0809.010515.
**39**	Wang, Q.; Byrd, K. A.; Navin, C.; Thilsted, S. H.; Try, V.; Kim, M.; Lejeune, M.; Worobo, R.; Than, S.; Fiorella, K. J. Nutrient Composition and Microbial Food Safety of a Locally-Processed Fish Product in Cambodia. Aquat Ecosyst Health Manag 2022, 25 (3), 73–81. https://doi.org/10.14321/aehm.025.03.73.
**40**	Tao, Z.; Sato, M.; Zhang, H.; Yamaguchi, T.; Nakano, T. A Survey of Histamine Content in Seafood Sold in Markets of Nine Countries. Food Control 2011, 22 (3–4), 430–432. https://doi.org/10.1016/j.foodcont.2010.09.018.
**41**	Douny, C.; Mith, H.; Igout, A.; Scippo, M.-L. Fatty Acid Intake, Biogenic Amines and Polycyclic Aromatic Hydrocarbons Exposure through the Consumption of Nine Species of Smoked Freshwater Fish from Cambodia. Food Control 2021, 130, 108219. https://doi.org/10.1016/j.foodcont.2021.108219.
**42**	Samy, E. Regional Survey of Histamine in Fish and Fish Products: Cambodia.; Bangkok, Thailand, 2008. Available at: https://repository.seafdec.org/handle/20.500.12066/4404 [Accessed 26 June. 2023].
**43**	Ngy, L.; Yu, C.-F.; Takatani, T.; Arakawa, O. Toxicity Assessment for the Horseshoe Crab Carcinoscorpius Rotundicauda Collected from Cambodia. Toxicon 2007, 49 (6), 843–847. https://doi.org/10.1016/j.toxicon.2006.12.004.

**44**	Zhu, H.; Yamada, A.; Goto, Y.; Horn, L.; Ngy, L.; Wada, M.; Doi, H.; Lee, J. S.; Takatani, T.; Arakawa, O. Phylogeny and Toxin Profile of Freshwater Pufferfish (Genus Pao) Collected from 2 Different Regions in Cambodia. Toxins (Basel) 2020, 12 (11), 689. https://doi.org/10.3390/toxins12110689.
**45**	Ly, D.; Mayrhofer, S.; Schmidt, J.-M.; Zitz, U.; Domig, K. J. Biogenic Amine Contents and Microbial Characteristics of Cambodian Fermented Foods. Foods 2020, 9 (2), 198. https://doi.org/10.3390/foods9020198
**46**	Ngy, L.; Tada, K.; Yu, C.-F.; Takatani, T.; Arakawa, O. Occurrence of Paralytic Shellfish Toxins in Cambodian Mekong Pufferfish Tetraodon Turgidus: Selective Toxin Accumulation in the Skin. *Toxicon* **2008**, *51* (2), 280–288. https://doi.org/10.1016/j.toxicon.2007.10.002.

Supplementary Table S3

Summary of evidence reported by the studies included in the systematic review.

Name of hazard	Name of food/source	Sampling location	Sampling point	Level of detection	Reference (Paper No. in S 2)
Astrovirus	Bats	Ratanakiri, Stung Treng, Preah Vihear	Farm	5.45%	11
Biogenic amines	Fishery products	Kandal, Kampong Som/Sihanoukville, Battambang, Kampong Chhnang, Kampong Cham, Kampong Thom, Siem Reap, Phnom Penh	Fishpond, processing site, market	8.1-2035 ppm	40, 41, 42, 45
Biotoxins	Fishery products	Coastal area, Kandal, Phnom Penh, Sihanouk Ville, Kratie	Nature, processing site,	0.04-463 ppm	43, 44, 46
Brucella spp.	Cattle and swine	Takeo	Slaughterhouse	0.15	3
Campylobacter spp.	Poultry, duck, cattle	Kampong Cham, Battambang and Kampot	Farm	5-80.9 %	8, 26
Clostridioides difficile	Fishery products	Battambang, Kampong Chhnang, Kampong Cham, Kampong Thom and Siem Reap	Markets	0.2%	14
Escherichia coli	Fishery products, poultry, pork	Phnom Penh, Bantaey Meanchay, Siem Reap	Slaughterhouse, market	Not detected (ND)-89.5%	4, 20, 32, 39
Faciola gigantica	Cattle and buffalo	Kratie, Takeo, Kampong Cham, Kampong Speu, Kampong Thom,, Pursat, Battambang, Kampong Chhnang, Prey Vang	Farm	0.1-16.37%	1, 5
Faciola spp.	Cattle and buffalo	Pursat and Kampong Speu	Farm	20%	16
Gnathostoma spinigerum	Edible frog	Phnom Penh, Pursat and Takeo	Market	ND-60%	9
Hepatitis E Virus	Pig/Pork	Phnom Penh	Farm	12.15%	37
Nipah virus	Bats	Phnom Penh, Battambang, Kampong Cham, Kandal, Prey Veng, and Siem Reap	Nature, restaurant	0.71-11.5%	18, 19, 38
Parasites[1]	Pig/Pork	Kandal, Kampong Speu, Kampong Thom, Preah Vihear, Ratanakiri, Phnom Penh, Stung Treng	Slaughterhouse, farm,	ND-73.7%	2, 7, 28, 30, 35
Parasites[2]	Fishery products	Battambang, Kampong Chhnang, Phnom Penh, Takeo, Kandal, Kratie, Pursat, Kampong Cham, Kampong Thom, Prey Veng, Siem Reap, Stung Treng	Market, villages, nature	0.25-100%	10, 12, 13, 14, 15, 17, 23, 24, 27, 29, 31, 33, 34, 36

[1] Evidence of parasites in pig/pork included *Taenia* spp., *Taenia soleum*, *Trichinella* spp., *Blastocystis* spp., *Ascaris* spp., *Entamoeba* spp., *Balantidium coli*, *Trichuris suis*, *Oesophagostomum* spp., *Metastrongylus* spp., *Gnathostoma doloresi*, *Capillaria* spp., and *Ascarops* spp.,

[2] Evidence of parasites in fishery products included *Opisthorchis viverrini*, *Haplorchis yokogawai*, *Pygidiopsis cambodiensis* n. sp., *Stellantchasmus falcatus*, *Gnathostoma spinigerum*, *Procerovum* sp., *Haplorchis pumilio*, *Centrocestus formosanus*, *Echinostoma mekongi*, and *Angiostrongylus cantonensis*

Salmonella enterica	Fish, poultry, pig/pork	Phnom Penh, Banteay Meanchay, Siem Reap	Slaughterhouse, processing site, market	3-100%	4, 6, 22
Salmonella spp.	Fish, poultry, pork, cutting boards	Phnom Penh, countrywide	Market	3.5-88.2%	25, 26, 39
Sarcocystis species	Cattle and buffalo	Cambodia	Market	100%	21
Staphylococcus aureus	Poultry, pork, cutting boards	Countrywide	Market	11.3-38.2%	25
Vibrio species	Fishery products	Siem Reap	Processing site	92.8%	39
